# Challenges and Opportunities of Targeted Behavioral Interventions for Groups at Risk for Developing Rheumatoid Arthritis

**DOI:** 10.3390/healthcare9060641

**Published:** 2021-05-28

**Authors:** Alessandra Zaccardelli, Jeffrey A. Sparks

**Affiliations:** 1 Division of Rheumatology, Inflammation, and Immunity, Brigham and Women’s Hospital, 60 Fenwood Road, Boston, MA 02115, USA; azaccardelli@bwh.harvard.edu; 2 Department of Medicine, Harvard Medical School, 25 Shattuck Street, Boston, MA 20115, USA

**Keywords:** rheumatoid arthritis, prevention, behaviors, lifestyle, interventions, risk

## Abstract

*Background*: Rheumatoid arthritis (RA) is a serious autoimmune disease which causes painful, swollen joints and can impact quality of life and increase morbidity and mortality. There are several preclinical stages of RA that correspond to at-risk groups that include: genetic risk, risk from behaviors, elevation of RA-related autoantibodies, and early clinical disease manifestations such as undifferentiated arthritis. Early interventions are crucial to slowing progression to and potentially preventing RA onset. Modification of behaviors among at-risk individuals may decrease RA risk. There are several challenges and opportunities in implementing preventative behavioral interventions, which may vary within different at-risk groups. *Methods*: We performed a narrative review of the literature, including meta-analyses focused on RA risk-related behaviors as well as publications investigating the potential efficacy of behavioral modifications on RA risk. *Results*: There are multiple behavioral risk factors associated with RA, including smoking, obesity, low physical activity, low quality diet, and poor dental hygiene, which may contribute to progression to clinical RA. Meta-analyses have been performed for smoking, excess body weight, and physical activity. Likelihood of adopting behavioral modifications may increase as RA risk increases. *Conclusions*: Clinicians may be able to tailor preventative approaches to various RA at-risk groups to help reduce RA risk, but further research is needed. A better understanding of the relationship of behaviors with RA risk and optimized approaches to implementing behavioral changes may allow for clinicians to tailor their preventative approaches for at-risk individuals.

## 1. Introduction

There are several phases of development of RA prior to full clinical onset. Groups within these risk phases may have different likelihood of RA onset. Individuals in certain risk groups may be targets of preventative approaches for RA. These RA at-risk groups include: genetic risk, behavioral risk, systemic autoimmunity (presence of RA-related biomarkers), and presence of clinical RA-related disease manifestations. Though the risk of RA in the general population is low, presence of these independent risk factors increases disease risk, which compounds when combined with other risk factors. Early behavioral modifications and preventative measures, even before systemic autoimmunity and joint involvement [1], are crucial to prevent rheumatoid arthritis progression. Understanding of risk, perceptions about the disease, and preferences regarding preventative measures may influence decisions regarding uptake and adherence of particular lifestyle changes. By evaluating each RA at-risk group and the potential promotors and barriers for preventative changes, clinicians can better understand how to tailor efficient targeted preventative therapeutic approaches.

## 2. Methods

For this narrative review, we conducted a literature review by searching PubMed for English-language research articles, meta-analyses, and review papers using various combinations of the following keywords: rheumatoid arthritis, risk, lifestyle, behavior, prevention, perspectives. We also searched for meta-analyses investigating established behavioral risk factors for RA including: smoking, obesity, high BMI, low physical activity, diet (alcohol, fish, omega-3 polyunsaturated fatty acids, red meat, caffeinated beverages, and sugar), and poor dental hygiene. We searched the bibliographies of all identified research articles, meta-analyses, and review papers. We also searched the American College of Rheumatology Abstract Archives to identify recent research studies.

## 3. Findings

### 3.1. RA At-Risk Group 1: Behaviors

Though the absolute risk of developing RA in the general population is low, with an incidence of RA in the general population of below 0.5/1000 per year [2], certain individuals may be at higher risk for developing RA due to behavioral patterns. Case-control studies in the general population have found that behavioral risk factors include cigarette smoking [3], inhalant-related occupations [4], obesity [5,6,7,8], low physical activity [9], low-quality diet [10], poor dental hygiene [11], maintenance of an abnormal microbiome [12,13], and lack of treatment for psychologic conditions like PTSD [14] and depression [15], habits which confer varying degrees of RA risk [16] (Table 1). These behaviors may also interact with other RA risk factors, such as genetics, to increase overall RA risk [16].

Smoking is the best-established risk factor for RA and may contribute to disease progression through gene-environment interactions by inducing localized mucosal inflammation in the airways and other anatomic sites, protein citrullination (a post-translational modification from arginine to citrulline), and downstream formation of anti-citrullinated protein antibodies leading to RA autoimmunity [17,18,19,20,21,22,23,24,25]. Ever and current smoking strongly increase the risk for RA compared to never smoking in both men and women [26,27,28,29] and anti-citrullinated protein antibody (ACPA)-positive and -negative RA [27]. There is an established dose-response for smoking and RA risk; while 1–10-pack years confer moderate risk, smoking above a threshold of 20-pack years strongly increases RA risk [3,26]. Other noxious inhalants like air pollution, silica, and pesticides may also contribute to pulmonary mucosal inflammation and RA development, indicating that lifestyle factors such as inhalant-related occupations may also contribute to RA risk [4].

Excess weight has also been shown to increase RA risk, though the mechanism of risk induction remains less clear. Excess weight increases RA risk overall [5,6,7,8,28,29], but when stratified by sex the association seems to be strongest among women [5,7]. Several studies have also found that increased adiposity confers an increase in RA risk per every 5 kg/m^2^ body mass index (BMI) increase, particularly in women [30].

High physical activity may reduce RA risk by reducing production of inflammatory biomarkers including C-reactive protein [31] and via an overall anti-inflammatory effect [31,32]. A meta-analysis of physical activity demonstrated that high levels of physical activity reduce RA risk compared to low levels, and that physical activity decreases RA risk compared to inactivity/occasional physical activity [32].

There is extensive literature on poor diet and RA risk, showing that intake levels of individual items such as high levels of red meat [33,34], low quantities of omega-3 polyunsaturated fatty acids [35,36,37,38] and fish [10,38], high caffeine intake [39,40], and low intake of fruit, vegetables, and vitamin C [41], as well as overall low-quality diet [42] increase the risk for RA.

Poor dental hygiene may confer additional RA risk as a consequence of periodontal disease and specifically, *Porphyromonas gingivalis*, the pathogen responsible. *P. gingivalis* expresses the peptidylarginine deiminase (PAD) enzyme, which is involved in protein citrullination that may lead to a loss of immune tolerance and RA [11].

Fostering an atypical oral and gut microbiome with high levels of *Prevotella copri* may increase RA risk [12,13]. Findings of sequence homology between *Prevotella* epitopes and RA-related autoantigens suggest that molecular mimicry may play a role in RA induction, but the mechanism remains unclear [12].

High levels of PTSD and depression symptoms may lead to chronic dysregulation of the hypothalamic–pituitary–adrenal axis and increased levels of systemic inflammatory markers, including interleukin-6 and C-reactive protein, which may be elevated prior to RA onset [14,15]. Therefore, behavioral patterns like lack of treatment for mental health conditions may increase RA risk.

There is also a risk of multiple lifestyle factors and RA development. For instance, while smoking, low alcohol intake, and increased BMI each moderately increase RA risk (population attributable risks of 15%, 12%, and 9%, respectively), individuals with all modifiable risk factors have a 41% risk of developing RA [43]. In addition, overweight individuals with a history of smoking have a 60% risk of developing RA [29]. At the same time, individuals with seropositive arthralgias adhering to 4 or 5 components (compared to none) of a healthy living index score consisting of never smoking, moderate alcohol use, normal BMI (18.5–24.9 kg/m^2^), regular exercise, and healthy diet showed a significant decrease (35%) in overall RA risk, indicating potential avenues for behavioral modifications to prevent RA [44].

Several observational studies have investigated the effect of behavior modifications on RA risk. Smoking cessation is the best studied behavioral modification to attenuate RA risk [1,45,46,47] (Table 2). For instance, compared to current smokers, former smokers who quit less than five years ago showed moderately diminished RA risk, and this was greatly reduced in those who quit 30 or more years ago [46]. Smoking cessation is also associated with a 20 year latency period of RA development [48]. Other behavioral modifications have also been studied. For instance, previous studies evaluating weight change and RA risk found that maintaining weight may reduce RA risk in comparison to weight loss or gain [49], but this may be due to the early rheumatoid manifestation of cachexia in individuals with undiagnosed RA. However, bariatric surgery was not associated with a reduced RA risk, so more work is needed to establish the role of weight loss in RA risk [50].

Dietary improvements may also reduce RA risk. Increased intake of individual substances such as alcohol may protect against RA [51]. Similarly, increased dietary beta-cryptoxanthin through consumption of items such as orange juice may protect against inflammatory disorders like RA [52]. In addition, meeting a healthy eating threshold composed of factors like increased consumption of vegetables, fruits, whole grains, nuts, legumes, long-chain omega-3 fatty acids, and polyunsaturated fatty acids as well as decreased consumption of sugar-sweetened beverages, red meats, processed meats, and trans fats, can reduce RA risk [53].

In addition, higher levels of physical activity have been shown to be associated with lower RA risk, with a 33% lower RA risk for >7 h of recreational activity per week compared to <1 h of physical activity per week [9]. It is also possible that behaviors like tooth brushing and flossing, which may decrease *P. gingivalis* as well as promote a healthy oral and gut microbiome, may reduce RA risk, but this has not been studied.

Thus, lifestyle modifications may decrease the likelihood of developing RA in the behavioral risk group. However, given the perception of RA in the general population, it may be challenging to encourage these types of behavioral modifications for RA prevention. Understanding RA perceptions within the general population can help advance potential interventions and public health initiatives to reduce RA risk conferred from behavior alone (Table 3). A previous study assessing disease literacy within the general population conducted interviews among individuals with no personal connection to RA and demonstrated an overall lack of RA knowledge [53]. Though some participants identified it as a disease affecting the joints, most were unsure about the cause of RA, incorrectly considering RA as a degenerative process [53]. Meanwhile, others speculated about a potential genetic component and some even believed that diet may contribute to RA progression, indicating that a subset may understand behavioral risk factors. Overall, interviewed individuals expressed a need for more public information about RA [53].

These findings especially reported limited knowledge about RA, ambivalence surrounding lifestyle changes to curb RA progression, and hesitancy to seek early clinical care, presenting challenges in initiating preventative behavioral changes. Other challenges include the development, implementation, and sustainability of behavior-modifying interventions.

**Figure 1:**
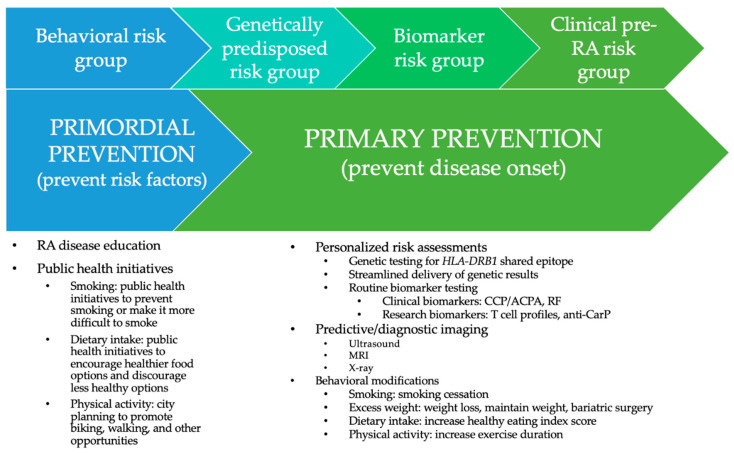
Primordial and primary prevention behavioral opportunities related to rheumatoid arthritis.

At the same time, there are opportunities to educate the general public on RA risk factors and outcomes (Figure 1). Targeted interventions like smoking cessation, weight loss, dietary supplementation, increasing physical activity, and good dental hygiene have already been conducted or proposed. Though adherence and willingness are unclear, there may be opportunities including educational initiatives and personalized risk assessments to help individuals in the general population to better understand RA risk so that they can make behavioral adjustments.

There exists a myriad of opportunities in encouraging behavioral changes, including personalized risk assessment and education tools to provide individuals with a better understanding of their risk, educate them on impacts of specific behavioral changes, and alleviate concerns surrounding predictive assessments (Table 3). These could be tailored to meet the needs of various risk groups. Our group conducted a randomized controlled trial called the PRE-RA Family Study, which incorporated a web-based Personalized Risk Estimator for RA (PRE-RA) tool [54,55,56,57] to provide a RA risk score based on individuals’ genetics, autoantibodies, demographics, and risk behaviors such as height, weight, physical activity, diet including fish and fish oil supplements, dental health, and smoking [54]. RA risk is presented in an interactive webpage displaying a thermometer with non-modifiable risk factors and modifiable risk factors that participants could adjust to see a visual representation of how the change would impact their risk based on the strength of the association and the direction of the modification [54]. The tool also includes personalized tips, text, and websites as well as a graphical representation of personalized relative risk of RA, and a percentage of lifetime risk for developing RA [54]. This format is likely easier to understand in comparison to traditional risk communications which many at-risk individuals find difficult to interpret [58].

The randomized controlled study compared standard-of-care RA education consisting of non-personalized risk education, the PRE-RA tool, and the PRE-RA tool plus motivational interviewing (PRE-RA Plus) to investigate the association between various educational interventions and RA knowledge [54]. Randomization to the PRE-RA and PRE-RA plus arms was associated with higher RA knowledge scores immediately after intervention, which was maintained during follow-up compared to standard of care. Educational intervention with the PRE-RA and PRE-RA Plus tools was associated with an increased ability to identify these RA risk factors which persisted throughout follow-up in comparison to the standard of care [54]. Perhaps more importantly, the PRE-RA tool increased motivation to improve RA-risk-related behaviors [55]. Subjects who received PRE-RA were more likely to increase ladder scores (RR 1.23 [1.01–1.51]) than those who received standard of care. In addition, PRE-RA subjects reported increased fish intake, tooth brushing, flossing, and smoking cessation compared to those who received standard of care [55]. This demonstrates that personalized medicine may improve likelihood of making behavioral modifications. Though some interviewed individuals expressed anxiety about learning their genetic risk, which may prevent them from pursuing preventative testing [59], personalized education via the PRE-RA tool was shown to decrease anxiety and concern for RA development [57], perhaps making them more likely to seek the preventative help they need. The trial investigating the PRE-RA tool may have had limited generalizability as the majority of subjects were highly educated. This also indicates that it may be difficult to recruit at-risk groups for educational interventions.

Given the potential hesitancy to make preventative RA-related lifestyle changes in low risk groups, other opportunities include public health initiatives, which can impact a large portion of the population; for instance, the regulation of smoking through taxes and age restrictions, or banning menthol cigarettes which were previously aggressively marketed to underprivileged communities. Public health initiatives could also discourage low-quality dietary items while encouraging healthier food groups. Other options include encouragement of physical activity via alternative transportation initiatives like increasing urban access to bicycles or city planning with more bike lanes. Many of these behaviors are already considered healthy for reasons besides RA, so there could be positive benefits for RA risk reduction even if not enacted for this reason. These would target the population at large under the pretext of broad health improvement brought on by healthy life choices and as a byproduct could reduce RA risk.

Behavioral modifications can be broken down into primordial and primary interventions, which can work to prevent RA-related behaviors and disease progression, respectively. Primordial interventions may be especially useful among the general population.

### 3.2. RA At-Risk Group 2: Genetics

For individuals with a genetic predisposition, there is an increased risk for RA which may be exacerbated by behavioral risk factors. Genetic risk includes a family history of RA, especially having a first-degree relative (FDR; parent, sibling, or child) with RA, which confers a 2- to 3-fold increased likelihood of developing RA compared to the general population. FDRs also express increased levels of rheumatic regional pain syndromes and RA-related autoantibodies such as ACPA and rheumatoid factor (RF) in comparison to the general population, which may be indicative of even further progression to clinical RA [60]. Other genetic risk factors include presence of the *HLA-DRB1* “shared epitope”, which is the strongest genetic risk factor for RA, particularly when combined with smoking and other environmental factors. Specifically, aberrantly formed citrullinated proteins are presented to T cells via the HLA-DRβ1 protein, which may have the downstream effect of antibody production by B cells [17,18,19,20,21,22,23,24,25]. Amino acid haplotypes at positions 11, 71, and 74 of the HLA-DRβ1 protein greatly increase RA risk and this risk is exacerbated by smoking [27,61]. FDRs who have the shared epitope are even more likely to express RA-related autoantibodies than FDRs without additional genetic predisposition. Other genes such as *PTPN22* (protein tyrosine phosphatase non-receptor type 22) also likely have causal roles in RA risk, and the mechanisms are being investigated [62]. Furthermore, 98 genes at 101 risk loci have been identified and demonstrated to increase RA risk [63]. Interestingly, studies finding RA monozygotic twin concordance at 15% suggest that genetics alone confer a limited risk for RA development [64].

Genetically predisposed individuals, especially those with pre-clinical RA manifestations, may be more amenable to preventative behavioral changes. In addition to smoking cessation, weight loss, increased physical activity, and dietary modifications, individuals with a family history of RA may benefit from other lifestyle decisions, such as consumer genetic testing for the shared epitope. When interviewed, many FDRs agreed that heredity is related to RA risk however most did not recognize behaviors like poor dental health or smoking as risk factors, and there was a low knowledge of personal behaviors, obesity, or poor diet and RA risk [54,55,56,57]. Individuals in several studies indicated that they would prefer behavioral to pharmaceutical interventions [59,65,66,67,68], expressing that they would be more likely to take preventative measures if they were at a substantially increased risk for RA (above 30% in the next five years), and if the prevention methods had high efficacy [68,69].

There may be educational opportunities which will have a significant impact in the population of FDRs to patients with RA, who may be more receptive to educational materials, especially considering the promising results of the PRE-RA study [54,55,56,57]. Tools like the PRE-clinical Evaluation of Novel Targets in RA website (www.preventra.net) may also help FDRs to assess and better understand their risk for RA. Since interviewed individuals expressed a preference for lifestyle modifications over medications as well as a desire to learn more about their disease [58], FDRs may be amenable to receiving RA risk assessments. This may be beneficial in providing at-risk individuals with more information to make decisions about their behaviors and lifestyle.

In terms of perceptions regarding behavioral changes, interviewed FDRs demonstrated similar beliefs as those in the general population. Many were unsure about the extent of their RA risk [58], expressed a lack of knowledge when making decisions about RA and RA risk, and were unwilling to make lifestyle changes without more information [59]. Many also mentioned that negative perceptions of RA may decrease the likelihood of seeking predictive help. Furthermore, they were specifically concerned about genetic testing, expressing concerns about accuracy and reliability of genetic testing [59] as well as fear of anxiety provoked by results of genetic tests [58,59]. Many also expressed the desire for a more personalized risk assessment to help them make informed decisions [59], or additional support to better understand risk and cope with psychological impact of receiving risk information [58].

Additional challenges surrounding genetic risk include communicating genetic risk information in a way that promotes disease understanding without causing anxiety [69]. For instance, it may be beneficial to provide genetic risk communications via trained geneticists using a streamlined/abridged protocol in a face-to-face or phone setting, rather than via mail or email only. It may also be useful to present genetic information graphically. There may be additional risks when using direct-to-consumer genetic testing services which may increase concern and anxiety [70]. Educational initiatives like the PRE-RA tool may be an important step in letting at-risk individuals know their likelihood of developing RA and encouraging relevant behavior changes (Figure 1).

### 3.3. RA At-Risk Group 3: Elevated Autoantibodies

Individuals who test positive for RA biomarkers including ACPA, RF, and research biomarkers such as T-cell clones [71], anti-carbamylated protein (anti-CarP) and anti-peptidyl-arginine deiminase type-3 (anti-PAD3) antibodies [72], may also be at increased risk for RA or exacerbated disease activity and joint damage. The association between ACPA and RF positivity and RA is well established [73]. There is a dose-response between increased ACPA levels and likelihood for developing RA. ACPA positivity is more prevalent among FDRs (3–6%) than in the general population (1%), and among smokers, women, and individuals between 45 and 55 years old [74]. Recent research investigating T cell profiles among genetically at-risk individuals, specifically FDRs, demonstrated that T cell clones were present prior to clinical onset of RA and increased from asymptomatic FDRs to symptomatic FDRs to RA disease patients. Thus, T cells increase over time throughout the pre-clinical RA period, especially in stages closer to RA onset [71]. Anti-CarP and anti-PAD3 levels were measured among RA patients and were associated with higher disease activity, particularly in association with other RA risk factors such as RF and ACPA. This indicates that Anti-CarP and anti-PAD3 may play a role in RA development and contribute to disease progression. Elevation of biomarkers like ACPA alone can increase risk of RA to about 5% and this association is even stronger when combined with other risk factors, especially family history of RA, increasing risk to 69% [2]. Antibody positivity is an important indicator of who may progress to RA and the level of association of lifestyle choices and RA risk as many risk factors are associated with autoantibody-positive RA. Prevention among this group may help to slow disease progression as well as attenuate disease manifestations if clinical RA occurs.

As these individuals have been seen by researchers or clinicians and likely been informed of their results, they may be more amenable to preventative behavioral modifications. Understanding their level of disease knowledge, satisfaction with disease education and risk communication, and desire to prevent clinical RA could help to develop targeted lifestyle interventions (Table 3). Interviewed individuals expressed low knowledge about RA and dissatisfaction with the lack of information on the efficacy of behavioral changes and RA risk, indicating that the current standard of care is inadequate in preparing high-risk individuals to understand and manage their RA risk [67]. Further, many expressed a specific anxiety about genetic testing, a potential barrier to this form of behavioral modification. Compared to asymptomatic individuals, symptomatic biomarker-positive individuals were more likely to express interest in behavioral changes, which is a challenge, as early prevention is thought to be essential to stopping disease progression. Similar to the genetic risk group, biomarker-positive individuals exhibited a preference for lifestyle modifications and a desire to learn more about personalized risk factors. At the same time, they expressed a desire to gain more information, assurance, and confirmation. Opportunities include a more robust educational tool to help these individuals better understand their risk and make decisions regarding their behaviors (Figure 1).

### 3.4. RA At-Risk Group 4: Clinical RA Features

Individuals in the clinical at-risk group include those who present with clinically suspect arthralgias, palindromic rheumatism, undifferentiated inflammatory arthritis, and extra articular inflammatory manifestations such as interstitial lung disease but without classifiable RA. Progression from systemic autoimmunity to clinical pre-RA manifestations such as inflammation and tissue damage may be due to mechanical stress and stimulation of mesenchymal cells, leading to chemokine release and monocyte attraction, as well as osteoclast activation, producing the classical articular presentation [75]. Individuals with pre-RA disease manifestations are at very elevated risk for developing classifiable RA. Some of these individuals may be appropriately treated by clinicians for RA, even if research classification criteria are not entirely fulfilled. Thus, the interpretation of whether these individuals are at risk for RA or already have it may be highly individualized related to the patients’ and providers’ shared goals and decision making.

Interviews conducted among individuals with clinically suspect arthralgias provide insight into perceptions surrounding RA development [76]. Though none of the interviewed individuals identified themselves as patients, they did mention social and physical consequences as a result of their pain and arthralgias. These individuals often express increased disease anxiety related to their symptoms. Many participants expressed concern regarding RA progression and uncertainty about the consequences of their prognosis. However, many participants adopted preventative behavioral changes after learning of their disease risk, specifically, dietary changes, haptonomy, yoga, tai chi, meditation, and mindfulness. These lifestyle changes promote health and reduce pain, highlighting that individuals with RA symptoms may be more likely to adopt behavioral changes to prevent classifiable RA [76]. Thus, it is possible that symptomatic individuals are more likely to make behavioral preventative changes than asymptomatic RA at-risk groups (Table 3). In this group, prognostic models may be essential to accurately provide risk estimations.

Thus, an opportunity among the clinical at-risk group may be encouraging predictive testing to forecast RA or inflammatory arthritis (IA) progression (Figure 1). For instance, ultrasound may be a useful predictor for individuals with ACPA-positivity and non-specific musculoskeletal symptoms, clinically suspect arthralgia, and palindromic rheumatism, but less accurate among others sub-groups [77]. Similarly, a model was developed to combine the severity, number, and locations of subclinical inflammation to determine different risk scores, with increasing RA risk based on increasing presence of inflammation [78]. Another model found that MRI-detected tenosynovitis was associated with IA and RA development, but that foot MRI scans did not increase predictive accuracy [79].

Predictive models may help to better understand the compounding risk from multiple risk factors leading to the production of personalized risk evaluations to more accurately predict RA risk. Individuals with combined risk factors may be at increased risk for disease. Similarly, individuals with a positive family history, high genetic susceptibility, smoking, and increased BMI had a 22-fold increased risk for ACPA-positive RA [80]. Additional predictive models have been developed for individuals in later phases of RA progression. For instance, a tool subclassifying individuals’ ACPA positivity and non-specific musculoskeletal symptoms and IA progression based on RA-related variables found that many individuals presented with non-specific musculoskeletal symptoms and no individuals with a low-risk score progressed to IA, while 31% of those with a moderate risk developed IA and 62% of individuals with a high risk progressed to IA [81]. Meanwhile, a prediction rule for RA development in seropositive (ACPA and/or IgM RF-positive) arthralgia patients categorized patients into 3 risk groups based on risk variables including FDR status, alcohol non-use, duration of symptoms < 12 months, presence of intermittent symptoms, and antibody status. with 82% accuracy [82]. Individuals in the intermediate risk group may have a 4.52-fold increased risk for RA, while those in the high-risk group have a 14.86-fold increased RA risk [82]. At the same time, genetic risk and pre-clinical risk factors may be combined to more accurately assess which clinically suspect arthralgia patients will progress to IA. A study evaluating clinically suspect arthralgia patients determined that IL-7R and IGF-1 were differentially expressed among individuals who did and did not progress to IA [83].

## 4. Conclusions

There are several behavioral factors which are associated with increased likelihood of developing RA, and which can be modified to reduce risk. As individuals’ RA risk increases from behavioral risk only to genetic, autoantibody, and pre-clinical RA risk, it may be possible to tailor strategies to encourage RA education and prevention. As risk increases, individuals may be more likely to make preventative behavioral modifications. However, early measures are more likely to effectively attenuate risk. In the general population, large-scale public health initiatives may be necessary to promote healthier lifestyle, with the byproduct of decreasing RA risk. In the genetic, autoantibody, and pre-clinical RA at-risk groups, personalized risk scores and education may be beneficial in helping individuals cope with their RA risk and make appropriate decisions regarding preventative care. More research is needed to determine the efficacy of specific behavioral modifications and likelihood of recruitment and long-term adherence to these lifestyle changes.

## Figures and Tables

**Table 1:** Meta-analyses of behavioral risk factors for rheumatoid arthritis.

Behavioral Risk Factor and Comparisons	Sex	Direction of Association	RR or OR for RA (95% CI)	Author (Year) of Reference
**Smoking**				
1–10 pack-years/>20 pack-years/>40 pack-years vs. never smoker	All	↑	RR 1.26 (1.14–1.39) RR 1.95 (1.65–2.27) RR 2.07 (1.15–3.73)	Di Giuseppe (2014) [3]
High (>40 pack-years) vs. low (never smoker)	All *	↑	RR 2.47 (2.02–3.02)	Di Giuseppe (2014) [3]
Ever vs. never	Men	↑	OR 1.89 (1.56–2.28)	Sugiyama (2010) [26]
			RR 1.47 (1.29–1.68)	Ding (2021)
Current vs. never	Men	↑	OR 1.87 (1.49–2.34)	Sugiyama (2010) [26]
			RR 1.27 (1.09–1.48)	Ding (2021)
Past vs. never	Men	↑	OR 1.76 (1.33–2.31)	Sugiyama (2010) [26]
Ever vs. never	Men *	↑↑ *	OR 3.02 (2.35–3.88) *	Sugiyama (2010) [26]
Current vs. never	Men *	↑↑ *	OR 3.91 (2.78–5.50) *	Sugiyama (2010) [26]
Past vs. never	Men *	↑↑ *	OR 2.46 (1.74–3.47) *	Sugiyama (2010) [26]
Ever vs. never	Women	↑	OR 1.27 (1.12–1.44)	Sugiyama (2010) [26]
Current vs. never	Women	↑	OR 1.31 (1.12–1.54)	Sugiyama (2010) [26]
Past vs. never	Women	↑	OR 1.22 (1.06–1.40)	Sugiyama (2010) [26]
Ever vs. never	Women *	↑ *	OR 1.34 (0.99–1.80) *	Sugiyama (2010) [26]
Current vs. never	Women *	↑ *	OR 1.29 (0.94–1.77) *	Sugiyama (2010) [26]
Past vs. never	Women *	↑	OR 1.21 (0.83–1.77)	Sugiyama (2010) [26]
>20 pack-years vs. never smoker	Men	↑	OR 2.31 (1.55–3.41)	Sugiyama (2010) [26]
>20 pack-years vs. never smoker	Women	↑	OR 1.75 (1.52–2.02)	Sugiyama (2010) [26]
**Excess weight**				
Obese/overweight vs. normal	All	↑	RR 1.31 (1.12–1.53) RR 1.15 (1.03–1.29) RR 1.21 (1.02–1.44) RR 1.05 (0.97–1.13) RR 1.32 (1.11–1.54) RR 1.08 (1.00–1.15) RR 1.23 (1.09–1.39) RR 1.12 (1.04–1.20)	Qin (2015) [6] Feng (2016) [5] Zhou (2018) [7] Feng (2019) [8]
Obese vs. normal	Women	↑	RR 1.26 (1.12–1.40)	Feng (2016) [5]
			RR 1.40 (1.24–1.57)	Zhou (2018) [7]
Obese vs. normal	Men	NS	RR 0.83 (0.65–1.05)	Feng (2016) [5]
			RR 0.89 (0.01–1.77)	Zhou (2018) [7]
Obese vs. normal	All **	↑ **	RR 1.47 (1.11–1.96) **	Feng (2016) [5]
Per 5 kg/m^2^ BMI increase	All	↑	RR 1.11 (1.05–1.18)	Ohno (2020)
			RR 1.09 (1.04–1.15)	Ding (2021)
Per 5 kg/m^2^ BMI increase	Women	↑	RR 1.15 (1.08–1.21)	Ohno (2020) [30]
Per 5 kg/m^2^ BMI increase	Men	NS	RR 0.89 (0.73–1.09)	Ohno (2020) [30]
**Physical Activity**				
Highest vs. lowest	Men and women	↓	RR 0.79 (0.72–0.87)	Sun (2021) [32]
Physically active vs. inactive/occasional active	Men and women	↓	RR 0.85 (0.79–0.92)	Sun (2021) [32]
**Dietary intake**				
Fish: 1 to 3 servings per week vs. never	Men and women	↓	RR 0.76 (0.57–1.02)	Di Giuseppe (2014) [10]
**Periodontitis**				
Periodontal disease (disease vs. no disease)	Men and women	↑	OR 1.97 (1.68–2.31)	Railson de Oliveira Ferreira (2019) [11]

BMI, body mass index; NS, non-significant; RA, rheumatoid arthritis; RR, relative risk; OR, odds ratio; CI, confidence interval; * Rheumatoid factor-positive RA; ** Seronegative RA.

**Table 2:** Studies investigating behavior modifications to reduce RA risk.

Behavior Modification	Sex	Direction of Association	HR or RR for RA (95% CI)	Author (Year) of Reference
**Smoking cessation**				
Quit ≥ 30 years vs. quit < 5 years	Women	↓	HR 0.63 (0.44–0.90)	Liu (2019) [45]
Quit > 15 years, quit > 1 year, current smoker	Women and men	↓	RR 0.70 (0.24–2.02)	Di Giuseppe (2013) [46]
**Bariatric surgery**				
Bariatric surgery vs. no bariatric surgery	Women and men	NS	HR 0.86 (0.54–1.38)	Maglio (2020) [49]
Alternative Healthy Eating Index score * adherence				
≥75 points vs. <75 points	Women	↓	RR 0.43 (0.27–0.67)	Marchand (2020) [52]
≥75 points vs. <75 points	Women **	↓ **	RR 0.41 (0.22–0.74)	Marchand (2020) [52]
≥75 points vs. <75 points	Women ***	↓ **	RR 0.47 (0.25–0.91)	Marchand (2020) [52]

BMI, body mass index; NS, non-significant; RA, rheumatoid arthritis; HR, hazard ratio, RR, relative risk; OR, odds ratio; CI, confidence interval. * Alternative Healthy Eating Index is composed of 11 food/beverage/nutrient groups and provides a score that ranges from 0 (least healthy) to 110 (most healthy) points. ** Seropositive RA *** Seronegative RA.

**Table 3:** Challenges and opportunities for behavioral modifications among rheumatoid arthritis risk groups.

At-Risk Group	Challenges	Opportunities
General population	Low general understanding or motivation related to RA Potential unwillingness to change behaviors Low absolute risk for RA	Education about RA, risk factors, early symptoms, and prevention strategies Targeted behavioral interventions Public health initiatives for other reasons may impact RA risk (legislations against smoking, legislation to promote healthy eating, etc.)
Genetic risk (e.g., *HLA-DRB1* shared epitope)	Unclear cost-benefit of testing for genetics Interpretation of direct-to-consumer genetic testing Risk of anxiety about genetic results	Education about genetic risks Personalized risk assessments Increased genetic testing and precautions when delivering genetic results
Family history (FDR without RA)	Perceptions of RA decrease likelihood of seeking predictive help Lack of information when making decisions about risk Potential unwillingness to make and sustain lifestyle changes Concerns about accuracy of predictive testing Concerns about anxiety of predictive test	Education about risk factors, early symptoms, and prevention strategies Personalized risk assessments Increased genetic testing and precautions when delivering genetic results
Biomarker risk (CCP+, RF, anti-CarP, anti-PAD3)	Lack of understanding of RA risk Need more information on RA and risk Anxiety about test results	Improve self-efficacy and health literacy Build on existing willingness to make lifestyle changes Build on expressed interest in gaining information, assurance, confirmation of meaning of clinical test
Clinical pre-RA risk (e.g., palindromic rheumatism, arthralgias, undifferentiated IA)	Some do not view themselves as patients or already feel they have RA Difficulty in understanding statistical risk of progression to RA Fear of pain, uncertainty of pain progression, developing functional limitations and prognosis	Education about early symptoms and risks Personalized risk assessments Present information in a way that makes sense to patients Learning risk group made risk groups more likely to adopt healthier habits changes, haptonomy, yoga and mindfulness.

RA, rheumatoid arthritis, FDR, first-degree relative.

## Data Availability

No new data were created or analyzed in this study. Data sharing is not applicable to this article.
